# Income security and infant health: Social context matters

**DOI:** 10.1073/pnas.2201818119

**Published:** 2022-08-08

**Authors:** Shervin Assari, Paula M. Lantz

**Affiliations:** ^a^Department of Family Medicine, Charles R. Drew University of Medicine and Science, Los Angeles, CA 90059;; ^b^Gerald R. Ford School of Public Policy, University of Michigan, Ann Arbor, MI 48109

Troller-Renfree et al. (1) recently published results in PNAS from an innovative study that randomly assigned 1,000 low-income mother–newborn dyads into two study arms: a large versus nominal unconditional monthly cash transfer. The results reveal increased brain development at 1 y, using resting electroencephalography, among the infants in the larger cash transfer group. This experimental study advances our understanding of the relationship between socioeconomic resources and health, specifically in regards to children’s brain development. Previous longitudinal observational studies have revealed similar findings but could not claim causal effects (2).

The authors (1) urge caution in using their results for public policy design, primarily because the intervention was only given to newborns for a 12-mo period. In addition, we would like to raise another important consideration for the design of income security policies for child health/welfare: A growing body of research demonstrates that, while income, education, and wealth are the fundamental social drivers of health, their benefits do not accrue in the same ways across racial groups (3). Simply put, there appears to be an interaction between socioeconomic status and race in regards to a wide variety of health outcomes across the life course, including chronic illness and mental health. Further, these “diminished health returns” to increases in socioeconomic resources appear to be operating through mechanisms at the neighborhood, system, and policy levels.

Although a strength of the Troller-Renfree et al. (1) study is that the majority of participants are racial/ethnic minority dyads, children in the larger cash assistance group were more likely to be Black, which could introduce a bias, as income differentially correlates with the brain development of Black versus White children. In the largest brain development study conducted to date (ABCD study), income shows stronger associations with the brain function and structure of White versus Black children at age 10 y (4, 5). Income has also been positively linked to depression for Black boys ages 12 y to 17 y (6). And, in another study, Black boys from high-income families who lived in predominantly White areas became more depressed over time (7). A proposed hypothesis for these and differences in the relationship between income and health across racial groups is increased exposure to interpersonal and structural discrimination (8).

Policy makers need to be aware that race-based stratification and discrimination in society means that the benefits of increased income security are not identical across racial groups, particularly for Black individuals. The root explanations for this phenomenon are social, economic, and political rather than biological. More income does not necessarily mean the same level of increased access to the environmental factors and goods/services that matter for health. For example, Black children are more likely to live in food deserts, and, therefore, additional monthly income may not similarly increase access to nutritional foods that are core to brain development (9).

Public policies that increase income security for children living in poverty also need to reform the systems of education, housing, food, healthcare, and policing that limit what additional income brings for families of color whose daily exposures and experiences are qualitatively different because of segregation and systemic racism (10).
